# A Qualitative Study of Perceptions, Strengths, and Opportunities in Cardiometabolic Risk Management During Pregnancy and Postpartum in a Georgia Safety-Net Hospital, 2021

**DOI:** 10.5888/pcd19.220059

**Published:** 2022-10-27

**Authors:** Kaitlyn K. Stanhope, Anna Newton Levinson, C. Taé Stallworth, Sophie Leruth, Emma Clevenger, Margaret Master, Anne L. Dunlop, Sheree L. Boulet, Denise J. Jamieson, Sarah Blake

**Affiliations:** 1 Emory University School of Medicine, Atlanta, Georgia; 2 Emory University Rollins School of Public Health, Atlanta, Georgia

## Abstract

**Introduction:**

Despite the strong link between cardiometabolic pregnancy complications and future heart disease, there are documented gaps in engaging those who experience such conditions in recommended postpartum follow-up and preventive care. The goal of our study was to understand how people in a Medicaid-insured population perceive and manage risks during and after pregnancy related to an ongoing cardiometabolic disorder.

**Methods:**

We conducted in-depth qualitative interviews with postpartum participants who had a cardiometabolic conditions during pregnancy (chronic or gestational diabetes, chronic or gestational hypertension, or preeclampsia). We recruited postpartum participants from a single safety-net hospital system in Atlanta, Georgia, and conducted virtual interviews during January through May 2021. We conducted a content analysis guided by the Health Belief Model and present themes related to risk management.

**Results:**

From the 28 interviews we conducted, we found that during pregnancy, advice and intervention by the clinical care team facilitated management behaviors for high-risk conditions. However, participants described limited understanding of how pregnancy complications might affect future outcomes, and few described engaging in postpartum management behaviors.

**Conclusion:**

Improving continuity and content of care during postpartum may improve uptake of preventive behaviors among postpartum patients at risk of heart disease.

**Summary**

**What is already known about this topic?**

Hypertension and diabetes during pregnancy are associated with increased heart disease risk. Less than one-half of the people with hypertension or diabetes during pregnancy receive guideline-compliant postpartum care.

**What is added by this report?**

Postpartum participants described barriers to managing and monitoring high-risk conditions postpartum, including competing priorities, such as finances, and lack of obstetric or gynecologic knowledge.

**What are the implications for public health practice?**

Additional support from the obstetric care team may improve postpartum care engagement for obstetric patients in a Medicaid-insured, safety-net population.

## Introduction

Cardiometabolic heath conditions are critical determinants of perinatal risk. Pregnant women with chronic diabetes or hypertension are at increased risk of infant and maternal morbidity (1–3). Additionally, incident cardiometabolic dysfunction during pregnancy (eg, gestational hypertension, preeclampsia, or gestational diabetes) is associated with elevated perinatal risk (2). Pregnant women with incident or chronic cardiometabolic dysfunction require monitoring during pregnancy to prevent and mitigate potential adverse outcomes (4–6).

After delivery, women who experienced cardiometabolic dysfunction during pregnancy are at elevated risk of severe maternal morbidity, postpartum complications, and future development of cardiovascular disease, diabetes, and hypertension (7–9). To detect and prevent complications, the obstetric care team should screen postpartum patients for ongoing hypertension or glucose intolerance in the postpartum period, counsel them on heart disease risk and prevention, and refer them to primary care for ongoing surveillance and management (10). However, less than one-half of patients with cardiometabolic complications of pregnancy receive guideline-concordant postpartum blood pressure or glucose screening (11–13).

Engagement in beneficial postpartum health behaviors can mitigate risks related to cardiometabolic conditions. Returning to or attaining a healthy weight, lactation, and control of glucose and blood pressure are evidence-based strategies to reduce future heart disease risk and might also reduce risk in a future pregnancy (14–16). If patients are unaware of their heart disease risk or prevention strategies, however, they may be less likely to engage in optimal behaviors. Limited data suggest that people have limited knowledge about the link between pregnancy complications and future disease risk (17–19).

Limited data exist on patient understanding and management of cardiometabolic risks during pregnancy, particularly in Medicaid-insured low-income populations, in which maternal morbidity and mortality are highest (20). By using the Health Belief Model (21), we sought to understand how (22), our study’s goal was to understand how postpartum participants perceive and manage risks related to an ongoing cardiometabolic disorder during and after pregnancy.

## Methods

### Study design

Our study consisted of in-depth interviews with low-income postpartum patients at a safety-net hospital in Georgia and was part of a larger study to develop, implement, and test a postpartum planning intervention for patients at high risk of severe maternal morbidity. We received approval for this study from the Emory University Institutional Review Board (STUDY00001427).

### Participants

Patients for this study were recruited from a single safety-net hospital system in Atlanta, Georgia, (Grady Hospital) and were eligible if they 1) were within 3 to 6 months postpartum after delivering a liveborn infant from October 2020 through January 2021; 2) received prenatal care in the Grady Health System; and 3) had a prenatal diagnosis of diabetes, chronic hypertension, a hypertensive disorder of pregnancy (HDP), inclusive of gestational hypertension or preeclampsia), or gestational diabetes. We identified potentially eligible patients through diagnostic codes in the electronic medical records and contacted them by telephone to invite them to participate. We conducted purposive sampling of postpartum participants who both had and had not attended their postpartum visit. Interviews were conducted during January through May 2021. Participants provided written informed consent before participating and were given a $50 gift card after interview completion.

### Data collection

We developed a semistructured interview guide to assess how patients understood and managed cardiometabolic risk conditions during pregnancy and postpartum (Table 1). After developing a draft of the guide, we shared it with members of our community advisory board, which consisted of local maternal health leaders, and revised the language according to board feedback. We then piloted the guide with 3 initial interviews, making slight modifications to language, and developed probes after each pilot interview.

**Table 1:** Key Domains of the In-Depth Interview Guide With Selected Questions Related to Understanding and Managing Cardiometabolic Risk Conditions, Atlanta, Georgia, 2021

Domains	Selected questions
**Prenatal**	
Pregnancy discovery	Tell me about when you found out you were pregnant.
Prenatal care	What were your interactions with providers like during prenatal care visits? What concerns did you have about your own health during pregnancy? How did the provider address those concerns?
Expectations for postpartum care	What did your provider tell you about follow-up care for your diagnosis?
Understanding and managing cardiometabolic risk conditions	What was it like to be pregnant with high blood pressure? Where else did you get information about high blood pressure? What did your provider tell you about high blood pressure?
**Delivery**	
Delivery hospitalization	What instructions did the doctor or nurse give you about follow-up care for yourself?
Discharge	Did you have a visit for postpartum care or any other follow-up visit scheduled at the time you were discharged?
**Postpartum**	
Adjustment	Tell me how things went for you during the first few weeks after delivery. For many women, the first few weeks after delivery are an adjustment. What are some of the things you had to adjust to following this delivery?
Self-care: unmet needs	Is there anything that makes it challenging to take care of your own health?
Postpartum visit (barriers and facilitators or reasons for nonattendance)	What helps you take care of your own health?
Ideal care	What recommendations or advice did the provider give you about taking care of your own health?

Interviews lasted an average of 78 minutes and were conducted by using Zoom. One or 2 trained study team members conducted each interview, and a team member took detailed notes. Interviews were recorded with the permission of the participant.

### Analysis

All interview recordings were professionally transcribed. We used MAXQDA (VERBI GmbH Berlin) for data management and transcript analysis (23). A directed content approach for coding data was guided by research questions (24). We developed a codebook by using a team-based approach in which all members read an initial 5 transcripts, wrote analytic notes with initial interpretations of the text, and developed candidate deductive and inductive codes. We applied the initial codebook to transcripts in teams of 2 and iteratively updated the codebook to produce a final codebook that was applied to the remaining data. Further identification of patterns across and within data were used to develop themes during a final stage of interpretation.

### Theoretical framework

We used the Health Belief Model to organize and interpret thematic results about participant management of pregnancy and chronic disease during pregnancy and postpartum and presented key themes by element of the Health Belief Model and timing (pregnancy or postpartum) (Figure). A study team member mapped and coded segments related to understanding and managing cardiometabolic risk conditions and general health to applicable elements of the Health Belief Model (threat, benefits, barriers, self-efficacy, and cues to action) and identified patterns across and within data to develop themes.

**Figure:**
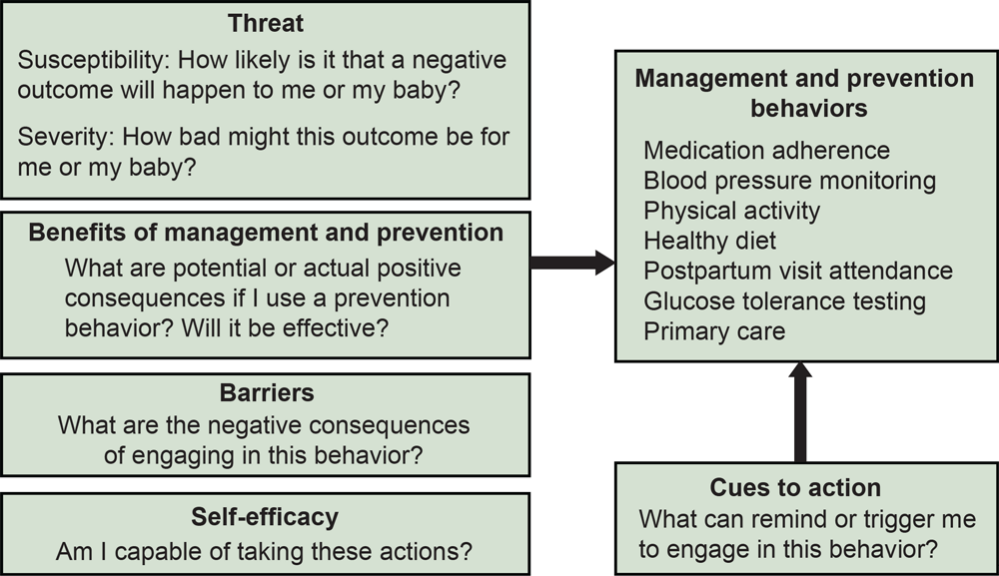
Diagram of the constructs of the Health Belief Model (22), as applied to the current study of high-risk cardiometabolic conditions during pregnancy and postpartum, adapted from (24).

Health Belief Model asserts that people make choices about their health risk behaviors depending on their perceived susceptibility to an adverse outcome, their perceived severity of the outcome, and their perceived benefits from and barriers to a given behavior (22,24). Perceived self-efficacy and external cues to action facilitate uptake of health-promoting behaviors. This framework helps identify possible opportunities for health care providers to improve patient support systems for managing cardiometabolic risk conditions.

## Results

Of the 93 postpartum participants identified as potentially eligible through electronic medical records, 28 (30%) completed an interview. By design, all study participants had 1 or more cardiometabolic risk conditions complicating pregnancy as recorded in the medical record (Table 2). Fifteen (54%) had hypertension. An additional 12 (43%) had a gestational hypertension diagnosis. Many (13, 46%) of those with gestational or chronic hypertension developed preeclampsia. Diabetes was rare, only occurring in 2 participants. Six (21%) participants had gestational diabetes. Most participants (19, 68%) had at least 1 previous pregnancy, 24 (86%) identified as non-Hispanic Black, and 26 (93%) were insured by Medicaid during pregnancy. Participants ranged in age from 18 years to 42 years, with a median age of 27 years.

**Table 2:** Characteristics of 28 Participants Who Completed Interviews for Cardiometabolic Risk Perceptions, Strengths, and Opportunities During Pregnancy and Postpartum Study, Atlanta, Georgia, 2021

Characteristics^a^	Value^b^
**Health insurance**	
Medicaid	26 (93)
Uninsured	2 (7)
**Cardiometabolic risk condition**	
Diabetes	2 (7)
Gestational diabetes	6 (21)
Hypertension, pre-existing	15 (54)
**Hypertensive disorders of pregnancy**	
Gestational hypertension	12 (43)
Preeclampsia	13 (46)
**Race and ethnicity**	
Hispanic	7 (2)
Non-Hispanic Asian	7 (2)
Non-Hispanic Black	24 (86)
**Attended 4–12-week postpartum visit**	16 (57)
**Reported having a primary care physician**	43 (12)
**Median (25th–75th percentile)**	
**Age, y**	27 (23.5–33.0)
**Parity**	2 (1–4)
**Gestational age at entry into care, week**	12 (10–21)

a Categories can overlap (ie, a participant may have a chronic diabetes and a gestational hypertension diagnosis).

b All values are number (percentage) unless otherwise indicated.

### Thematic findings

#### Perceived susceptibility

Perceived susceptibility is a person’s belief of how probable or improbable a given adverse outcome is for them. We considered beliefs about susceptibility to be both the participant’s understanding of their pregnancy-related diagnosis and their perception of their risk for future adverse outcomes.

Almost all participants understood that they had a cardiometabolic risk condition during pregnancy (Table 3); however, understanding of their specific diagnosis varied. Although all participants with diabetes or gestational diabetes understood their diagnosis, 9 of 16 participants with HDP were unclear about their exact diagnosis. For example, one participant with HDP explained that her blood pressure was not of concern until a spike immediately before her pregnancy. Another one explained, “Since it wasn’t always persistent on high . . . next visit it would be a little bit lower. Then it’ll be high . . . About the last month is when it started staying consistent. So that’s when they was like, ‘Oh, I think we going to have to get him out ASAP.’*”*

**Table 3:** Key Themes and Quotes From 28 Participants Who Completed Interviews by Using Elements of the Health Belief Model and Perinatal Status, Atlanta, Georgia, 2021

Prenatal		Postpartum	
Subthemes	Postpartum quote	Subthemes	Postpartum quote
**Susceptibility**			
Few or no perceptible symptoms (for all hypertensive disorders and gestational diabetes)	**Attending participant^a^ with gestational hypertension and preeclampsia** All my vitals were always so stable. They just sent me the cuff in the mail just, just because, um, they wanted me to watch myself. But, no, it was not, . . . There wasn’t really any discussion. Um, all of the . . . going through prenatal, nothing was out the normal for my pregnancy. I didn’t have anything abnormal.	• Belief that pregnancy-related conditions would just go away and were not of concern following pregnancy • Perception that chronic hypertension (as diagnosed in the medical record) was only of concern during pregnancy (when it was detected with each pregnancy), likely because of limited engagement with care outside of pregnancy	**Nonattending participant^b^ with gestational hypertension** I wasn't really concerned about my blood pressure as much as probably other people may have been. I know it's something that runs in my family, but it has never been a problem that I had. There's more of something that seems to be just gestational.
**Severity**			
• Primary concerns: impact of high-risk condition on the developing fetus (stillbirth, miscarriage, preterm birth, macrosomia, low birth weight) • Limited concerns: stroke or maternal death (during delivery) because of hypertension	**Attending participant^a^ with gestational hypertension** Interviewer: And with, with having preeclampsia, any time during your pregnancy, were you ever worried about your health or your baby's health? Response: Yes, I did...Like would he have it? Would he be . . . Would he have high blood pressure? Like, would I have my baby too, too, too early? Because my mom, see, my mom had . . . [pre-eclampsia], my momma had me early. She had me about when she was about [6 months or 7] months.	• Potential stroke or death because of hypertension (the silent killer) and heart disease • Experiences with family, history of hypertension, and heart disease	**Attending participant^a^ with preeclampsia** When I went to my visits they was like, “Oh, your blood pressure’s up. You don't feel sick?” And it really put my perspective in like I say of how serious it was because I . . . know how serious it was, but to actually go through it with yourself and knowing these are dangers when someone's actually telling you, this is how serious it is in your pregnancy. You could be walking around fine . . . what they say, they call blood pressure, the silent killer, and your blood pressure be sky high and you could just pass away.”
**Benefits**			
• Primary: preventing potential adverse consequences for baby • Limited: maintaining one’s own health	**Participant with chronic hypertension and superimposed preeclampsia^c^** Interviewer: Do you feel like being pregnant made it easier to stop smoking or harder? Why? Response: Yeah, it was easier. Because I know why I had to. I had a, why . . . that [stopping smoking] was a challenge at first, but I was told that before I even got pregnant, so I wasn't surprised by that.	• Primary: seeing one’s kids grow up or staying healthy to care for family • Limited: heart disease prevention (1 participant only)	**Participant with gestational hypertension** Now I still take my iron pills and like every now and then I take an aspirin just like, you know, to be on the safe side 'cause I'm like, now I have 4 kids I have to watch over and I have to take care of myself.
**Barriers**			
• Medication side effects • Challenges remembering to take medication • Cost of blood pressure monitor • Time, particularly if working or caring for older children	**Attending participant^a^ with chronic hypertension and superimposed preeclampsia^c^** Yeah. I understand where they were trying to go. I got it because I was high risk, so I definitely needed to rest. [inaudible] but I have 2 other kids. I couldn't rest as much. Blood pressure medicine, no. I was taking . . . my last time taking blood pressure . . . they keep trying to prescribe it to me, but it makes me sick. I just can't, it makes me so sick.	• Time and energy to focus on own health while caring for infant and older children • Cost or availability of blood pressure monitor • Finding a primary care physician who will take uninsured or Medicaid-insured patients • Food as a source of comfort	**Nonattending participant^b^ with preeclampsia** I’m just now really getting to myself to be honest with you. . . It happened subtly and just unconsciously. I was just so focused on my kids, and the newborn requires so much intricate care. And I was just so . . . I threw myself into accomplishing that I think that it, like slowly things would slip. Like, oh, I didn't shower today. You know? And I don’t realize that till 8 o’clock at night, [laughs] at night. You know? **Attending participant^a^ with preeclampsia** The last time I called, which was about a month and a half ago, they didn't have nothing available. I'm trying to be a new patient. I'm really trying to get in, but it's hard.
**Cues to Action**			
• Structured diabetes care curriculum • Worksheets for glucose monitoring and structured counseling • Telephone calls for blood pressure checks • Alarms, pill organizers	**Attending participant^a^ with chronic hypertension, superimposed preeclampsia^c^, and gestational diabetes** Mainly what I did for myself was to try to set an alarm so that we know it's time to check your blood pressure, in the mornings make sure . . . And I had went and bought a pill organizer so I can keep my medicine by my bed and wake up and have the medicines right there. So, I wouldn’t have to look for them. . . I liked the educational process, to know what to eat and not to eat because, like I said, that was the first time anybody told me when I got pregnant what I should not eat and what I needed to slow down eating because I didn't know.	• Home blood pressure check • Postpartum visit (limited) • Family advice and support (eg, through babysitting) • Insurance brochures	**Attending participant^a^ with chronic hypertension with superimposed preeclampsia^c^** They suggested that I see a primary doctor about my blood pressure. Oh, not my blood pressure, but since I had gestational hypertension. . . [W]e go to the YMCA, and the Y, it accepts the Medicaid that we have so it be somebody there watching the children while we go exercising, . . . so like WellCare and another Medicaid, it pay for it for us. They pay for our exercising; they pay for people to watch the children while we go exercising. [Wellcare] be handing out brochures, but I never tried it out until like we went the other day. **Participant with chronic hypertension** Instead of just saying, Okay, I'm going to take a smoke. Go and take a walk instead. So, I've been hearing her in the back of my mind, “Come on, Ms. [last name]. You can do it. It's been working out really well though.”
**Self-Efficacy**			
• Home blood pressure and glucose monitoring • Higher among participants with known chronic conditions • Existing relationships with primary care	**Attending participant^a^ with chronic hypertension** My numbers were always great. They were never high. The only one time it did get high, I was actually in labor and didn't know. And that's the day that I went to the hospital. But, other than that, my numbers always stayed low.	• Understanding warning signs Home blood pressure monitoring, including at retail outlets with blood pressure cuffs	**Attending participant^a^ with gestational diabetes and gestational hypertension** My sugar may be regular. Sometimes it'd be either, or every now and then, it'd be both. Since I'm not consistent, and I'm not on no high blood pressure medication, I just take it and watch myself and try to record, so when I go back to the doctor, I'll let him see it. Interviewer: Taking it [medication] at home, how did that make you feel? Participant: Like I was trying to help take care of myself. I knew how disastrous high blood pressure could be if it went too high, what could happen to me.

a Attending participant: attended a postpartum visit within the Grady Health System after her recent pregnancy.

b Nonattending participant: did not attend a postpartum visit within the Grady Health System after her recent pregnancy.

c Superimposed preeclampsia: Preeclampsia that develops in a patient with existing hypertension.

Most (12 of 16) participants with HDP or gestational diabetes in the postpartum period believed their condition was no longer an issue. Additionally, 5 of 15 participants with hypertension were unconcerned about their blood pressure, stating that it was high during pregnancy but not otherwise of concern (Table 3). “They tried to give me some blood pressure meds, and I told them, ‘it’s normal, it will go away in due time.’ I know my body because that’s what happened with the last one,” one participant with hypertension said.

Patients who attended the postpartum visit (attendees) and those who did not (nonattenders) described similar concerns about complications, primarily about infant complications. However, 2 of 16 attendees described discussions with the postpartum visit provider about hypertension that helped them understand their own risk.

#### Perceived severity

Perceived severity is a person’s perception of potential danger from a given disease or an adverse outcome. We focused on participants’ perceptions of adverse consequences of their cardiometabolic disease diagnosis. Many (13 of 28) participants were concerned about risks to the developing fetus, such as a miscarriage or preterm birth, because of their cardiometabolic risk condition. A nonattender with gestational diabetes said, “Mainly, [gestational diabetes] can affect [the baby]. Sometimes, baby come out real, real small. Sometimes, people have miscarriage, [because of] it, and sometimes, your baby can come out real, real big.”

In contrast, 6 of 28 nonattender participants expressed concern over risks to their own health, primarily describing possible strokes either during labor or at any time. A nonattender with HDP said, “Because I was getting to the point where I could have had a stroke, a seizure, heart attack, because all that anger and my blood pressure . . . the doctor said just go ahead and induce [me]. Because if they leave the baby in there, it could either come to the baby or my life. So, I was like, wow. I started crying when she told me that, because I was like, I’m going to die.”

After pregnancy, 4 of 16 participants described concern about potential risk because of gestational diabetes or HDP. Eight of 15 participants with hypertension and diabetes expressed understanding of their continued risk, although this was not true for participants who believed their hypertension to be relevant only during pregnancy. Both postpartum visit attendees and nonattenders described similar levels of concern about high-risk conditions during pregnancy, primarily for the baby. Slightly more attendees (7 of 16) than nonattenders (4 of 12) described concerns for future risks.

#### Perceived benefits

Perceived benefits are perceived or actual positive consequences resulting from a given health behavior. During pregnancy, 6 of 28 participants described engaging in specific behaviors to prevent adverse infant outcomes or to optimize their own health during pregnancy by managing weight gain and glucose levels. Perceived healthy behaviors included glucose and blood pressure monitoring, insulin injections, and exercise. One participant with diabetes and HDP explained, “I mean, diet is a main thing and exercise. . . I walked like 30 minutes to 40 minutes every day. . . Yeah. It helps, because whenever I test on home, the sugar level was perfect, and on every visit, they saw the sugar level and all that, that I tested. So, it was good, and they told me to do the same thing, like diet, exercise.”

During the postpartum period, some participants described engaging in prevention behaviors such as following up with primary care, maintaining a healthy diet, and exercising to stay healthy and to take care of children. An attendee with hypertension explained, “I’m 26 now and I had my first child when I was 19, so when I was younger, I wasn’t thinking about stuff like that [primary care visits]. But now that I had the hypertension when I was pregnant and I’m getting older, I feel like I need to focus on that because I want to be here to see my kids grow up.”

A few (5 of 28) participants exercised, dieted, or followed up with primary care specifically to lose weight. A nonattender explained, “That was another thing, my weight. Oh, my goodness. I was not happy and I’m still not happy, but I joined the gym.” One participant described engaging in healthy behaviors (exercise, diet, and smoking cessation) to prevent heart disease (Table 3). Postpartum visit attendees and nonattenders described similar perceived benefits to prevention behaviors during and after pregnancy. However, more attendees (11 of 16) than nonattenders (3 of 12) described plans to engage in primary care following pregnancy.

#### Perceived barriers

Perceived barriers are perceived or negative consequences of a given behavior or costs associated with that behavior. During pregnancy, participants described medication side effects and costs as barriers to engaging in blood pressure monitoring or medication use. An attending participant with hypertension said, “They tried to give me some blood pressure meds, and I told them, ‘It’s normal,’ it will go away in due time. . . I’m not going to take it because it made me feel nauseous and it tires me even more than what I am.”

During postpartum, 6 of 28 participants identified childcare as a barrier to self-care, or participants prioritized their children’s needs over their own, which limited their ability to engage in self-care behaviors or to seek follow-up care. A nonattender with hypertension needed to remain at home to care for her preterm infant with a feeding tube, although her care team asked her to return to the hospital for readmission because of her dangerously high blood pressure, as measured at a home visit. “Just because of how high they said [my blood pressure] was that day, but I think he was still on the feeding tube at that time, so my other children, they don’t know how to change a feeding tube. It was just, I couldn’t go [to be readmitted] at that time.”

Additionally, 5 of 28 postpartum participants explained how financial and insurance barriers prevented them from attending needed follow-up for primary or specialty care after their pregnancy. Participants were limited in their choice of providers, because some providers did not accept Medicaid or uninsured patients. Primary care practices that accepted Medicaid had limited availability, and participants were often unable to find an available appointment. Other participants described similar barriers to engaging in prevention behaviors during and after pregnancy. Two nonattenders at postpartum visits described mistrust of doctors as a barrier to seeking primary care.

#### Cues to action

Cues to action are reminders or triggers to engage in health-promoting behaviors when a person is ready. During pregnancy, cues to action stemmed primarily from counseling, reminders, or check-ins from the clinical care team. For example, participants with diabetes or gestational diabetes reported receiving support through one-on-one counseling and check-ins with a trained nurse and materials and guidance on testing blood glucose. This counseling helped remind participants to monitor glucose levels. In contrast, participants with hypertension or HDP largely created their own systems for reminding themselves to take medication (eg, pill organizers, alarms). One attending participant with gestational diabetes and hypertension explained, “Mainly what I did for myself was to try to set an alarm so that we know it’s time to check your blood pressure, . . . and I bought a pill organizer so I can keep my medicine by my bed and wake up and have the medicines right there.”

After pregnancy, participants described few cues to action from the clinical care team to support heart health behaviors. Despite explicit probes, only 8 of 16 reported discussing relevant healthy behaviors at the postpartum visit. For example, in one interview, the interviewer asked if the attending participant with HDP was asked about blood pressure at all at her postpartum visit and if [the clinician] was aware that the participant had high blood pressure during pregnancy. The participant indicated a negative response and stated, “They don’t ask that there.” The participant went on to remark that when discussions did occur, participants valued them; however, in some cases, reminders from the provider were insufficient, given the barriers. For example, 12 of 16 attending participants said that their postpartum provider told them to follow up with primary care, but 5 had not yet done so because they could not find a childcare provider or did not have time in addition to the demands of childcare.

Participants also received advice and support from family as cues to action for postpartum behaviors, describing how family members counseled them on their diet, encouraged them to take walks or time for themselves (and watched children while they did), or reminded them to check their blood pressure. One attending participant with HDP and gestational diabetes explained, “Because she [participant’s mother] has high blood pressure and has been taking her own blood pressure for years, she knew . . . what was normal for me and what was not. . . When I came home and I had a headache, she encouraged me to take it, and at that time it was high.”

Finally, 2 participants reported that outreach from Medicaid encouraged them to engage in healthy behaviors (exercise and scheduling primary care visits). For example, one participant explained how she started exercising after receiving a brochure from her Medicaid provider stating that her Medicaid covered exercise and childcare at the YMCA (Table 3).

Postpartum visit attendees and nonattenders received similar cues to action during pregnancy but described differences postpartum. First, attendees were more likely than nonattenders to report having a primary care provider outside of pregnancy (12 of 16 vs 0 of 12). Second, all attendees with hypertension described counseling on blood pressure management at the postpartum visit, which nonattenders did not receive.

#### Self-efficacy

Self-efficacy is a person’s belief that they are capable of engaging in an action that will result in positive change. During pregnancy, 5 of 15 participants with a chronic condition expressed comfort and confidence in understanding and managing their condition. Similarly, multiparous participants who had an HDP diagnosis in a previous pregnancy described confidence in managing HDP in each of their recent pregnancies. Home glucose and blood pressure monitoring, paired with training on how to monitor them accurately and when to report results to a physician, gave participants a sense of control over their condition during pregnancy.

After delivery, 7 of 28 participants described how monitoring and understanding their blood sugar or blood pressure levels postpartum gave them confidence in managing their chronic conditions. An attending participant with HDP and gestational diabetes remarked, “Since I’m not consistent, and I’m not on high blood pressure medication, I just take it and watch myself and try to record, so when I go back to the doctor, I’ll let him see it.”

Participants who were already engaged in care for a chronic condition before pregnancy expressed confidence about managing their condition postpartum. In contrast, participants who had a pregnancy-induced condition or who did not understand their diagnosis did not express confidence in their ability to manage or follow up on their condition. Postpartum visit attendees and nonattenders did not vary in perceived self-efficacy for managing high-risk conditions.

## Discussion

We presented the narratives of 28 low-income postpartum women of color from a high-risk, safety-net hospital about their perceptions and understanding of their cardiometabolic risks during and after pregnancy. Applying the Health Behavior Model, we described successful pathways through which participants engaged in prevention and management behaviors. We also noted multiple opportunities to address barriers, improve cues to action, and facilitate optimal postpartum health for patients with cardiometabolic conditions. Beyond gaps in knowledge and clinical support postpartum, our findings demonstrate how structural barriers (childcare, insurance, transportation) must be addressed to improve postpartum and long-term health for people giving birth who have high-risk cardiometabolic complications.

Given the findings of our study and prior research (17–19), clinicians should expand and improve counseling related to cardiometabolic risk conditions during pregnancy, particularly around the need for future and ongoing surveillance and management. In our study, and prior studies, we found a disconnect between patient diagnoses and their own understanding of their pregnancy and future risk related to cardiometabolic disease (17–19,25). Consistent with prior research, participants in our study prioritized the needs of their developing fetuses and babies over their own health (17,26). Effective postpartum counseling may include value-based discussions helping mothers see heart disease prevention as part of caring for their family (27). Counseling that connects weight management, exercise, and diet to heart disease prevention may help motivate some people. In our study, only one participant explicitly connected her weight management goals to managing her hypertension. Our findings, however, showed that even when motivated, some participants were unable to overcome structural barriers to engage in healthy postpartum behaviors. Thus, innovative strategies are necessary to improve postpartum follow-up for patients with cardiometabolic complications of pregnancy in low-income populations. Strategies might include telehealth, home visiting, or specialty postpartum transition clinics following high-risk pregnancies (28). Clinics could also implement warm handoffs, in which a care coordinator assists the patient in identifying and contacting a provider to make an appointment for needed care (29).

The results of this study should be interpreted in light of its limitations. First, because of the diversity of diagnoses in our sample (by design), our guide did not probe all potential management behaviors for each diagnosis and asked open-ended questions (eg, we asked, what have you been doing to take care of yourself, rather than, do you take blood pressure medication). Thus, we are only able to base our analysis on information from the targeted questions asked during the interview, paired with information abstracted from the medical record. Second, the sample of 28 participants represents only 30% of potentially eligible patients. The low participation rate might reflect that the postpartum period is a busy time for most, or it might have been related to lingering effects of the COVID-19 pandemic. Finally, we did not systematically ask about postpartum care experiences in previous pregnancies, which might guide a person’s approach to condition management.

Improving understanding of the link between cardiometabolic complications of pregnancy and future heart disease risk can empower pregnant and postpartum women to better manage their own health. Study participants were interested in weight loss and disease management, but they received little guidance from their clinical care team, even when they attended the postpartum visit. Health systems must implement innovative strategies to support postpartum women, particularly those at high risk of severe maternal morbidity and future heart disease. In this low-income, Medicaid-insured population, few participants were engaged in care before pregnancy, and the postpartum period represents a unique opportunity to engage them in prevention and disease management (29). However, because of the many structural barriers noted, education or written referrals alone are insufficient. Successful strategies should both build on existing values, such as the desire to stay healthy for their family and address the demands of childcare and finances postpartum.
